# CANon: Lightweight and Practical Cyber-Attack Detection for Automotive Controller Area Networks

**DOI:** 10.3390/s22072636

**Published:** 2022-03-29

**Authors:** Youngmi Baek, Seongjoo Shin

**Affiliations:** 1 Department of Computer Software Engineering, Changshin University, Changwon 51352, Korea; 2 Resilient CPS Research Center, DGIST, Daegu 42988, Korea; sj_shin@digst.ac.kr

**Keywords:** in-vehicle network, sequential hash chain, one-time key, controller area network

## Abstract

Automotive cyber-physical systems are in transition from the closed-systems to open-networking systems. As a result, in-vehicle networks such as the controller area network (CAN) have become essential to connect to inter-vehicle networks through the various rich interfaces. Newly exposed security concerns derived from this requirement may cause in-vehicle networks to pose threats to automotive security and driver’s safety. In this paper, to ensure a high level of security of the in-vehicle network for automotive CPS, we propose a novel lightweight and practical cyber defense platform, referred to as CANon (CAN with origin authentication and non-repudiation), to be enabled to detect cyber-attacks in real-time. CANon is designed based on the hierarchical approach of centralized-session management and distributed-origin authentication. In the former, a gateway node manages each initialization vector and session of origin-centric groups consisting of two more sending and receiving nodes. In the latter, the receiving nodes belonging to the given origin-centric group individually perform the symmetric key-based detection against cyber-attacks by verifying each message received from the sending node, namely origin authentication, in real-time. To improve the control security, CANon employs a one-time local key selected from a sequential hash chain (SHC) for authentication of an origin node in a distributed mode and exploits the iterative hash operations with randomness. Since the SHC can constantly generate and consume hash values regardless of their memory capacities, it is very effective for resource-limited nodes for in-vehicle networks. In addition, through implicit key synchronization within a given group, CANon addresses the challenges of a key exposure problem and a complex key distribution mechanism when performing symmetric key-based authentication. To achieve lightweight cyber-attack detection without imposing an additive load on CAN, CANon uses a keyed-message authentication code (KMAC) activated within a given group. The detection performance of CANon is evaluated under an actual node of Freescale S12XF and virtual nodes operating on the well-known CANoe tool. It is seen that the detection rate of CANon against brute-force and replay attacks reaches 100% when the length of KMAC is over 16 bits. It demonstrates that CANon ensures high security and is sufficient to operate in real-time even on low-performance ECUs. Moreover, CANon based on several software modules operates without an additive hardware security module at an upper layer of the CAN protocol and can be directly ported to CAN-FD (CAN with Flexible Data rate) so that it achieves the practical cyber defense platform.

## 1. Introduction

Cyber-physical systems (CPSs) are commonly applied to critical infrastructure and future-oriented services enabling the quality of life to be improved. These systems promise enhanced efficiency, convenience, and safety by integrating physical and computational components tightly. Recently, the physical components have suffered from various cyber-attacks, which aim to disrupt the intended functionality of the CPS, such as Stuxnet [1]. Such attacks mainly exploit computational components to manipulate the characteristics of physical components. The manipulation for the control of the physical component, therefore, may lead to unexpected accidents and abnormal operations. For instance, in the case of the electric automotive CPS, an attacked actuator spins the connected motor quickly and slowly within a range that the driver is not aware of in abnormal situations. It leads to an increase in fatigue and, consequently, it may break down while driving. Since the security of the automotive CPS is directly connected to the safety of drivers and passengers, it is critical to protect them against a cyber-attack. Especially, nodes in the in-vehicle network for the automotive CPS cannot identify whether a certain node transmits its message or not because the contents of the transmitted message do not provide any information about origin. If an adversary (i.e., an infected node) falsifies and then transmits the control data that is required while driving, the ECUs as nodes control the driving based on the falsified data received. Therefore, when an internal node is infected through various routes from the outside, the infected nodes prevent safe operation by transmitting falsified control data that the driver in a vehicle does not anticipate. This paper allows legitimate nodes to filter CAN messages from this infected node and to use only safe messages as control data while driving.

In this paper, to ensure safe traveling control of automotive CPSs, we investigate an effective and reliable method to support the security service detecting cyber-attacks, considering technical challenges inherent in in-vehicle networks. In this regard, the three problems to be solved are formulated as follows:

- PROBLEM 1: What types of cyber-attacks can the proposed security service detect in real-time? it considers the cyber-attacks that can occur while driving.
- PROBLEM 2: What levels of security and safety can it provide while driving?
- PROBLEM 3: What is a suitable method to provide data integrity and availability for resource-limited nodes? With such a method, we should consider how to satisfy the requirements of real-time processing and timeliness for safety-critical applications such as the automotive CPS.
- PROBLEM 4: How do we minimize a key exposure problem when detecting cyber-attacks based on a symmetric key? We should address that either the same secret key used for a long period or the redistribution of new keys increases the potential for exposing security vulnerabilities when using a key-based detection scheme.
- PROBLEM 5: How do we design a new security service for the legacy in-vehicle network and the internal architecture in terms of a cost-effective system? We consider the ways to achieve scalability, feasibility, and adaptability of the proposed security service at the same time.

To achieve safe control by answering the problems above, we propose a novel practical cyber defense platform, which is called the controller area network with origin authentication and non-repudiation (CANon). It offers a lightweight and efficient detection method against cyber-attacks for the in-vehicle network by employing an origin authentication-based cyber-attack detection approach using a one-time symmetric key. To detect cyber-attacks, CANon is focused on identifying whether control messages transmitted from the origin are trustworthy or not while CANon is able to provide data integrity and availability for resource-limited nodes.

To conduct lightweight detection in real time, CANon applies a hash function to verify the origin of the message using an authentication tag. The resource-limited CAN nodes are sufficient to perform origin authentication within a given limited time while it provides a high level of security for transmitted messages. To ensure its efficiency, CANon is designed with a combined method of centralized-session management and distributed-origin authentication. In centralized-session management, a gateway node is responsible for managing designated groups and every session of each group. The origin authentication is distributed to all CAN nodes except for the gateway node. To reduce key exposure occurrences derived from key re-distribution, CANon does not distribute or share any secret key over the CAN bus. Instead of key distribution, CANon enables each node to generate a sequential hash chain only valid to a particular session and to randomly select a one-time local authentication key only valid to the individual transmission during the given session. Furthermore, CANon assigns the new state of being random to the sequential hash chain at the start of a given session for each group in order to improve security. To evaluate the performance of CANon, we conduct both theoretical security analysis and experimental analysis and evaluate the performance of CANon in terms of the robustness against cyber-attacks and the practicality of real-time processing. In the theoretical analysis, we examine the robustness of CANon against cyber-attacks in terms of hash collision probabilities as the length of a hashed key is varied. In the case of experimental analysis, an experimental environment is constructed with real CAN nodes of Freescale S12XF ECUs and virtual CAN nodes of CANoe.

The main contributions of our research are as follows. First, despite not mounting additional hardware modules for a new security service, the software-based security platform we design for resource-limited ECUs ensures a high level of security and operates very lightly and quickly. Second, CANon is a new security platform that runs on each ECU without modification of the legacy protocol for in-vehicle networks. In addition to that, this approach requires the existing ECUs to implement only a minimal security service and to deploy it to them easily. It indicates that CANon is designed considering scalability. Third, this is a meaningful attempt to apply a simple sequential hash chain structure practically alongside real-time cyber-attack detection regardless of any hash functions. The sequential hash chain with a set of secret keys is managed within the given group and the certain session. In addition, the strength of the proposed structure is the capability to offer secret keys constantly without explicitly sharing them. Therefore, due to this structure, a key chain’s length is not affected by the memory space of the ECU. Finally, to ensure that our approach is feasible, we implement the proposed CANon in a proof-of-concept prototype. It demonstrated that CANon not only enables in-vehicle communication in a reliable way by detecting cyber-attacks with origin-authenticity assessments, but CANon operating in vehicular ECUs is also lightweight with low computational complexity.

The remainder of this paper is organized as follows. In Section 2, we describe the background and challenges to be addressed when providing a new security service for in-vehicle networks, and then introduce our approach at an abstract level to achieve the goal of this paper. In Section 3, we present the overall design of CANon to perform one-time local key-based authentication. A theoretical security analysis is conducted to examine the security level of the proposed CANon in Section 4. In Section 5, we determine the proposed variables required for offering practicability and then evaluate the performance of the proposed platform. Finally, Section 6 provides the conclusions.

## 2. Background and Challenges

This section presents a brief introduction of the conventional CAN protocol and CAN bus to indicate its vulnerabilities. In addition, we discuss the assumptions and considerations of efficient cyber-attack detection as well as define a threat model in the in-vehicle network. We investigate previous studies for cyber-attack detection in CAN to highlight the difference between our methodology and other work.

To enhance safety and efficiency, modern automotive CPSs are already equipped with many electrical and electronic devices such as radar, ultrasonic, wheel speed sensors, and electronic control units (ECUs). In order to move toward full automation, the emerging advanced driver assistance systems need to be realized. In this regard, there is no doubt that more ECUs need to be equipped for safe driving, user convenience, and reliable control. Furthermore, the closed-loop systems of the modern automotive CPS are transformed into open-networking systems that can perform remote control functions and various networking-based information services. For instance, automotive manufacturers offer their own remote services to start, manage, and control vehicles [2,3,4]. They are commonly based on wireless networking technologies such as Bluetooth, cellular technology, and Wi-Fi [5,6]. The in-vehicle network is designed to have one or more gateways to connect to the external networks, though its architecture is more complicated [7,8]. This leads to automotive CPSs being exposed to new security vulnerabilities. There are many cases of cyber-attacks exploiting security vulnerabilities. Hyundai’s BlueLink was demonstrated to be vulnerable to security threats by security firm Rapid7 [9]. It used the smartphone and Wi-Fi to hack into the stationary vehicle and succeeded in starting the vehicle remotely. Chrysler’s vehicles have been hacked and controlled by a laptop more than 10 km away [10]. While driving, the music poured from the speaker and the windshield wipers suddenly worked regardless of the driver’s intention. The adversaries easily accessed the vehicles in order to take control of the vehicles. It indicates that in the in-vehicle network, not only are the plain data transferred between ECUs without encryption but also the unauthorized user is enabled access to the internal system without any restrictions through the gateway.

To enhance security and protect against the vulnerabilities of the in-vehicle communication systems, a security service including authentication, confidentiality, and data integrity is required. Nevertheless, recently, it has not been easy for the automotive CPS to offer explicit security services for in-vehicle networks. It should take a very cautious approach for the automotive CPS. In consideration of the uncertainty that will likely appear in terms of stability, safety, and reliability, it is not easy to change the complex and stable structure and sophisticated connected components. Moreover, in order to preserve cost-effective manufacturing, modern automotive CPSs are equipped with many ECUs with limited performance. They are insufficient to perform encryption and decryption for all contents of one control message in real-time. To support the heavy-computational function for confidentiality, additive hardware security modules with a high performance increase the additional cost and weight, as well as the complexity of manufacturing. Therefore, we address these technical issues by using a new software-based defense platform considering practicality and effectiveness.

### 2.1. Key Characteristics and Limitations of Controller Area Network

In this section, we provide a brief background of the automotive CPS and the communication protocol applied for the in-vehicle network. The automotive CPS mainly consists of three major segments: The powertrain, chassis, and body. To support the exchange of control data needed for traveling control in the major segments, several in-vehicle networks that exist independently have interconnected each other. This indicates that the in-vehicle network consists of local networks that can be connected through gateway nodes. A gateway node is responsible for forwarding the messages of one network to another network. Each local network is typically constructed using either the industry-standard controller area network (CAN), local interconnect network (LIN), or the recently developed FlexRay. In particular, CAN is mainly used for the body and powertrain [11].

**Figure 1:**
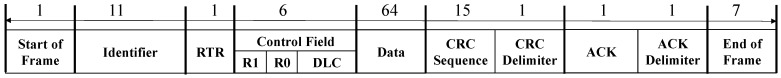
Standard format of a CAN 2.0A frame where the marked number indicating the length of each field is shown in bits.

CAN is a de facto standard and a widely supported network in the automotive industry, requiring minimal manufacturing costs, real-time processing, automation, and distributed processing. CAN is referred to as a network bus as well as a protocol. CAN is known as an efficient network with a bus topology in which all CAN nodes (i.e., ECUs) are physically connected by synchronizing the bit rate [12,13]. In addition, the CAN standard is widely known as a reliable communication protocol since it is capable of detecting a message collision and a bit error at a physical layer and autonomously rectifying colliding transmission. Using contention-based channel access, the CAN protocol broadcasts its frames containing vehicular control information. In CAN, a node checks the state of its bus with a busy state or an idle state. It tries to send a message only if it is in an idle state. There might be one or more nodes to broadcast their own messages on a common CAN bus. Although collisions are likely to occur on the CAN bus at that time, it has no effect on the operations of CAN. This is because CAN supports an arbitration mechanism called carrier sense multiple access/collision detection with an arbitration on message priority (CSMA/CD & AMP). Therefore, it performs reliably, transmitting a message to arrive in time for control. In other words, when two or more nodes simultaneously transmit frames, CSMA/CD & AMP at the physical layer arbitrates this collision in such a way that the node with the lowest value of the identifier (ID) field in its CAN frame wins. It indicates that the lowest value has the highest priority in transmissions. This arbitration mechanism controls all transmissions over the CAN bus at the physical layer. The ID field that presents the format of a CAN frame is shown in Figure 1.

After one node succeeds in broadcasting its frame, they determine whether to use or discard it whenever the other nodes receive it. The receiving nodes identify interesting messages among messages transmitted over the CAN bus by using the value of the identifier (ID) field of a CAN frame. They do not check who sent the interesting message. In particular, a high-speed CAN of 1 Mbps for event-driven communication is used for powertrain and safety-critical systems such as the engine control unit, anti-lock braking, and cruise control.

Although the classic CAN is designed to transmit an event-triggered message, the CAN frame can be transmitted either periodically or sporadically to ensure driving safety [14,15,16,17]. In powertrain and body systems, it is especially critical to transmitting messages periodically in order to ensure reliability, robustness, and real-time processing for vehicle control. In practice, the actual CAN nodes of a vehicle are designed to periodically broadcast their own messages over the CAN bus at a datalink layer. Instead, only designated nodes interested in the specific message will accept that data from their controllers. In that, the CAN communication of automotive CPSs operates as multicast communication where one node sends data to multiple nodes at once on a data layer. In this paper, a container with information about the different CAN designs specific to each manufacturer is referred to as CAN-DB (CAN-DataBase) [16,18]. Note that the contents of CAN-DB are useful for identifying the relationship between the sending and receiving nodes and periodicity for us, but manufacturers do not release the data of CAN-DB at all. In CAN-DB, a sending node may be designed to broadcast one or more messages over the bus. A receiving node can be designed to receive the designated messages that are transmitted from either the same sender or different senders.

As shown in Figure 1, there is no field to provide any information to identify both sending and receiving nodes since CAN performs message-based transmission using a message ID. It indicates that it has no capability to identify if a malicious source tampers with the transmitted data. Moreover, not only does it not provide enough space to convey security-related information but there is also no reserved field for security services in the CAN frame. For instance, the firmware for an ECU can be manually updated either with the help of an external hardware module that is connected directly to the OBD-II (On-Board Diagnostics version II) port or over the FOTA (Firmware Over-The-Air) using wireless connection technology. At that time, there is no doubt that there is still a high chance that stealthy attacks exist through a backdoor for app hackers and spyware in in-vehicle networks. Nevertheless, it does not have the ability to detect suspicious messages infiltrated through the external networks by an adversary in order to cause unintentional behavior during driving.

### 2.2. Assumptions and Considerations of Efficient Cyber-Attack Detection

In automotive CPS, cyber-attacks conducted through various external links can be broadly classified into two categories: An injection attack and a hijacking attack. The former cyber-attack aims to disturb the control ability to drive and the functions of a target system. To achieve it, an adversary can be connected to an internal CAN network through various external links, and then infect normal CAN nodes [19]. For instance, it can be connected directly to the in-vehicle network via physical interfaces such as OBD-II, disc, and USB. It also exploits a short-range wireless access method such as Bluetooth, radio frequency identification (RFID), dedicated short-range communication (DSRC), and RF-based remote keyless entry. Its physical access aims to cause abnormal operations while updating the ECU’s firmware. In terms of CAN, this type of cyber-attack can take place as a counterfeit message injection through a compromised ECU. In addition, it may be possible that the pre-programmed message is sent over the CAN bus after installing the adversary’s short code into an ECU. It is defined as an injection attack in this paper. The latter cyber-attack aims to steal and gain privileges to access the internal data and the components of a target system [20]. This cyber-attack aims to exploit threats through the theft of authentication and personalization information and illegal acquisition of the authorization of control service. It is called a hijacking attack in this paper. To conduct this hijacking attack on ECUs, an adversary needs to first analyze messages shared through the external network, namely vehicle-to-everything communication. To do this, it exploits long-range wireless access including broadcasting channels and addressable channels. The broadcasting channels include GPS, satellite radio, and traffic message channels, and the addressable channels indicate those of cellular data networks. Through them, the acquired information is used to infiltrate inside automotive CPSs, which eventually makes the injection attack possible. Regarding efficiency, the hijacking attack should be defended by the service providers that provide external networking services. Detecting this cyber-attack is outside the objectives of our paper. To ensure vehicle safety. especially for driving, this paper focuses on the development of a robust countermeasure against injection attacks.

In CAN, injection attacks can come in three forms: Modification, replay, and masquerade attacks. First, a modification attack modifies the contents of an existing message. Second, a replay attack transmits the existing message without any modification. Third, in a masquerade attack, an adversary pretends to be an origin node. In CAN, the masquerade attack has no choice but to be conducted by exploiting the same ID of the existing message. This is because the CAN protocol does not provide any information for the identification of an origin node and only identifies the ID of the transmitted message.

The most straightforward way to detect these cyber-attacks in CAN is to examine the forgery of the control data transmitted over in-vehicle networks. In other words, to identify the fact that the messages are not manipulated by any adversary regardless of its modification or masquerade attack, it needs to verify the data integrity of transmitted control data. One of the most effective ways to guarantee data integrity is to use cryptographic techniques to provide the confidentiality of information such as AES (i.e., symmetric encryption) and SHA-256 (i.e., Secure Hash Algorithms). However, resource-constrained ECUs in the in-vehicle networks might be not sufficient to perform encryption and decryption of the control data within a transmission time interval with the range of 5 ms to 100 ms [16,21,22]. Therefore, to implement heavy computational functions such as encryption, it is inevitable that additional processors are required to possess high performance [23]. To place additional hardware inside the automotive vehicle, it is necessary to re-design new internal architecture; hence, it inevitably leads to an increase in associated expenses. Therefore, in the CAN network, rather than applying encryption-based authentication to low-performance ECUs, we consider the way to achieve scalability and adaptability of a security service in terms of cost reduction, computational overhead, and complexity challenge.

As an alternative to the encryption method, there is the use of an authentication tag that utilizes some of a message, which is referred to as message authentication. Message authentication, which is commonly used to verify data integrity, is an efficient and proactive method to defend against data forgery attacks conducted through physical manipulation or comprised network connections [24,25]. Although message authentication does not provide the property of non-reputation, in this paper, message authentication aims to provide both data integrity and origin identification. We use the term message authentication as synonymous with origin authentication in CAN.

As mentioned above, when a malicious node impersonates a certain normal CAN node and then transmits the modified control data, the CAN protocol is unable to detect this cyber-attack in practice. In the literature, most of the studies providing an authentication service for CAN tackle the message-based transmission of CAN [26,27,28]. An authentication tag, which is referred to as a message authentication code (MAC), for message authentication is mainly generated by a secure hash function from a given original message [29,30,31,32]. This approach requires a symmetric key between sending and receiving nodes to generate and verify MAC [33,34,35]. Therefore, the level of security of this approach depends on how sophisticated and secure the distribution of the secret key used is.

The great strength of a symmetric key-based method, which is efficient in terms of processing cost and speed, is fascinating when applying it to CAN requiring real-time processing. Unfortunately, in CAN, there are challenges to be addressed in order to perform efficient cyber-attack detection via origin authentication using secret keys. The longer the secret key remains unchanged and is used for verification on the CAN bus, the more likely the secret key will be exposed. This means that adversaries can conjecture and discover the same secret key since they show the same pattern as many previously transmitted messages. In order to strengthen the security of the used symmetric key, it is essential to distribute new secret keys constantly. Therefore, the use of the symmetric key for origin authentication needs to support a mechanism for managing and distributing secure keys between a pair of CAN nodes. When performing origin authentication using symmetric keys, many studies employ different methods to periodically distribute new secret keys [36,37,38]. Designing a sophisticated key distribution mechanism, however, may require the sacrifice of real-time transmission to CAN nodes. For instance, if the secret key used between the sending and receiving nodes is newly distributed for every transmission, the transmission of a CAN frame that should be transmitted at a predefined schedule can be delayed due to the time that it takes to distribute the secret key between CAN nodes. This is because the key distribution needs to take precedence over data transmission. Using a secure dedicated channel to distribute a secret key requires the addition of a new physical communication channel to CAN. It will adversely affect the weight and wiring complexity of the vehicle.

In this paper, to tackle its limitations above, we apply the CAN’s multicasting nature to node grouping and conduct symmetric key-based authentication detecting cyber-attacks injected over the CAN bus while minimizing key exposure.

### 2.3. Conventional Cyber-Attack Detection Methods for Controller Area Network

In this Section, to exploit an origin authentication approach to detect various cyber-attacks accurately in CAN, we investigate the existing origin authentication methods. We introduce several approaches, which are suitable to the multicast communication environment of CAN, to authenticate the data origin efficiently [36,39,40,41,42].

One study focuses on a symmetric key-based authentication approach. Peirrig et al. have proposed a timed efficient stream loss-tolerant (TESLA) scheme in which a digital signature method is applied to source authentication [39]. It allows a symmetric key to be exposed to perform the delayed per-packet data authentication for the digital signature using the received packet sequence. Rather than sharing a secret key at the initial time point, they inform the packet of the secret key used transmitted at time t of a receiver after a given delay δ. In other words, a disclosed secret key $s\left(t\right)$ of the packet *p*$\left(t\right)$ received at time t is used to authenticate the packet *p*$\left(t-\delta \right)$ received at time t−δ (i.e., δ>0). It compares MAC of the received packet $r\left(t-\delta \right)$ with MAC generated using the disclosed key $s\left(t\right)$. The transmission efficiency is reduced due to additional delivery authentication information. To synchronize the nodes’ clocks, it should perform the designed initial protocol and set the parameters at the initial time, such as the key disclosure delay time, the interval duration time, and the session starting time among nodes. At the same time, it requires the initialization of a key chain. In addition, one packet should be divided into small pieces since it is unable to be inserted entirely into a CAN frame due to its size. This means that it takes longer than the expected time taken to authenticate one packet. As a practical matter, in CAN, the issues of delayed authentication, the large number of parameters to be initialized, and the complex dedicated initialization process are great challenges to satisfying real-time requirements.

In CAN, one study applies a group-based authentication approach. Groza et al. have proposed a lightweight broadcast authentication protocol for CAN, called LIBRA-CAN, based on a shared key within a given group [40]. They employ a strategy of two-stage authentication using both a data-dedicated frame with a message for control and an authentication-dedicated frame with multiple MACs. Therefore, LIBRA-CAN may consume a maximum of 50% in total CAN bandwidth to authenticate CAN nodes because one authentication frame is used for each data frame. In LIBRA-CAN, the role of CAN nodes is divided into a gateway as the master and a node as the slave. A master is responsible for distributing a secret key to slaves belonging to a given group, as well as performing the authentication. Each slave transmits its authentication frame after forwarding a data frame to the master. The authentication frame contains all MACs of slaves in the given group. Therefore, one master and its slaves can verify its data frame by using the contents of the transmitted authentication frame. In this regard, to overcome the limited length of a data field in a CAN frame, it takes preventive measures whereby the slaves’ MACs are split across multiple authentication frames. This may lead to an increase in the authentication delay with an increase in the size of a group. Therefore, the effectiveness of LIBRA-CAN depends on the length of the data field of the CAN frame, the size of a given group, and the number of groups. Furthermore, to manage different groups, LIBRA-CAN not only requires the gateway to be a high-computational-powered node but also the slaves to register themselves for a gateway before data communication.

There is another group-based authentication approach in which nodes vote on the redundancy of MACs for a group [41]. Voting means that all nodes within a given group should participate in cross-checking the messages transmitted over a network. Every member of the group sends its message with both its own MAC and the MACs derived from the messages transmitted by the other nodes in the group. All receivers vote to authenticate a sender’s message only when they obtain both the sender’s original message and the other members’ validities. Therefore, one node is authenticated by this method only after all nodes of the group transmit their messages with redundant MACs. This is not suitable for CAN since it causes an authentication delay that cannot be neglected. This method is designed without considering the time taken to perform the authentication in a node.

One research study places emphasis on lightweight operation through the combination of the group and symmetric key-based approaches. Kang et al. proposed a source authentication protocol based on a one-way hash chain in CAN [36]. In this protocol, a group shares secret keys during a given period. Receivers verify messages with the authenticator received from a sender. To address the key exhaustion of the one-way hash chain and consider the shortage of memory capacity of ECUs, they focus on minimizing the key generation delay by keeping a small number of keys for authentication. Against hash collision attacks, they have proposed a tree structure that hierarchically maintains a certain number of keys for authentication. Nevertheless, authentication suffers from the depletion of keys in the tree when a series of cyber-attacks continue to occur as frequently as the height of the designed tree.

One approach as tried to enhance key security by limiting the number of times the symmetric key is used [42,43,44]. Hammi et al. proposed a mutual authentication protocol for network association and data transmission [42]. They use a one-time password (OTP) for authentication. There are two types of devices: A device with the role of a coordinator and a device with the role of a member. A new device should first join the network before transmitting data. The coordinator uses the new device’s OTP to authenticate it. After the coordinator’s authentication, the coordinator uses that OTP to prove itself while delivering a secret key, which is required for the device to transmit the data, to the joined member. In this approach, it is assumed that both the device’s information and a pre-shared key are never exposed. To generate OTP, it uses the hash-based MAC (HMAC) algorithm from a pre-shared secret key. Although each device uses different OTPs during network association and data transmission, the series of OTPs used for each device never varies over time due to the use of the pre-shared secret key. In addition, they do not provide a certain key distribution to prevent masquerade attacks. This may also result in successful attempts to inject brute-force attacks under the assumption that an adversary has enough time.

### 2.4. Threat Model

Due to the exposed security vulnerabilities of the CAN bus, it is possible for an adversary to impersonate a legitimate node. The adversary aims to induce a risk of control over the automotive CPS by forging and transmitting messages as if it was a legitimate node and then letting receiving nodes use falsified messages to control driving. We define the capabilities of this malicious node on the CAN bus as follows. The adversary is unable to access any security information (e.g., a secret key and user processes) held in the physical memory of CAN nodes. However, due to the nature of the CAN bus, an adversary that is directly connected to the CAN bus can eavesdrop on all messages sent by legitimate nodes. It can also inject its forged messages according to the operations of the legacy CAN protocol. The adversary is capable of finding similar or identical patterns of the contents from transmitted messages by performing analysis of the eavesdropping on the messages over the CAN bus. Hence, by eavesdropping over a long duration, the adversary can construct CAN-DB containing the messages’ meta-information, including the IDs, lengths of control data, and transmission periodicities for each designated message. Consequently, the adversary injects various falsified messages based on the constructed CAN-DB into the CAN bus.

An adversary can conduct four cyber-attacks in the in-vehicle network: A replay attack, a modification attack, a masquerade attack, and a brute-force attack. These have been classified based on the transmission period, indicating the interval between the start of the consecutive message transmissions and whether the control data are tampered with. The amount of time representing the transmission period of the message sent by a legitimate node is denoted as the transmission time interval (TTI) in this paper. First, it is defined as a replay attack when the adversary transmits it again after storing one of the messages sent over the CAN bus. In particular, a replay attack does not modify the content of the control data, which is presented in the data field of a CAN frame, in the message. Regardless of the periodicity of a certain message sent by the legitimate node the adversary injects it into the CAN bus. Second, it is defined as a modification attack when the adversary transmits it after the stored message is modified. This attack is also injected regardless of the message’s transmission periodicity. In the modification attack, the content of the data field in the message is falsified. Third, when the adversary transmits the forged message with the TTI of the message of a legitimate sending node, it is referred to as a masquerade attack. Since the adversary wants to appear as a legitimate CAN node, the masquerade attack is conducted while exploiting the transmission periodicity of the message sent by the legitimate node. In addition, it intends to modify the contents of the data field of the legitimate message transmitted in order to provide the wrong information to the nodes and cause an unexpected driving state. The injection of the masquerade attack and the transmission of a legitimate node occur either simultaneously or around the scheduled TTI of the normal message. A receiving node has no choice but to believe that all of the received messages are legitimate CAN messages, even if they have different contents. It can also adroitly change the contents of other fields following the data field in order to cause a form error when the adversary transmits its own message with the value of the ID field of the normal message. Against these cyber-attacks, when either sending or receiving nodes detect the error, the retransmission mechanism of the CAN protocol is conducted automatically. Consequently, the targeted message is continuously retransmitted. This behavior, which is typically called a bus-off attack, intends for nodes to enter the bus-off state. At that time, if the transmission priority of the injected message is the highest, it can be referred to as a denial-of-service attack. Finally, a brute-force attack adopts a trial-and-error method to construct valid control data in the data field. If the adversary launches a brute-force attack by randomly organizing the contents of the data field, it can be injected regardless of the transmission periodicity of the legitimate message. At that time, it does not intend to create a new value of the ID field since any receiving node is not interested in that message if the value of the ID field is replaced with an arbitrary value that does not exist in CAN-DB. In this sense, the brute-force attack can include modification and masquerade attacks. Furthermore, in the same way as the masquerade attack, the brute-force attack can be injected in the way sense that it modifies the values of the control field, the CRC-related field, and the ACK field. It is regulated in the physical layer of CAN during transmission due to form errors in the CAN frame. Therefore, a brute-force attack for form errors is not a valid cyber-attack, but these cyber-attacks also contribute to increasing the bus load. Consequently, this paper limits the attack model to two types of cyber-attacks: A brute-force attack, including the modification of the contents of the data field as if the adversary was a legitimate node, and a replay attack without modification.

## 3. Cyber-Attack Detection Based on Origin Authentication for Controller Area Network

In this section, we propose a new cyber defense platform to detect various cyber-attacks flowing into in-vehicle networks from the outside, called CANon. It performs the detection of cyber-attacks injected through receiver-based origin authentication for one sending node of a given group. In addition, the origin of the given group is not allowed to repudiate the fact that it has transmitted its own message over the CAN bus. This is because receiving nodes of the given group verify every message transmitted by the sending node using its own information.

To provide a realistic and practical solution for the existing CAN platform, the design objectives of CANon are as follows. The first is to detect injected cyber-attacks accurately in real-time. To perform traveling control over the CAN bus, the term real-time means completing those instructions within the minimum TTI determined for CAN messages. Therefore, CANon provides legitimate control data for the nodes as well as reliable and safe driving for humans against replay attacks and brute-force attacks. The second is to achieve secure origin authentication using the symmetric key-based method while it achieves un-traceability and addresses the key exposure problem. The third is to reduce computation and communication costs by minimizing the process of session update, key generation, and origin authentication so that the operation of CANon does not disturb the operations of the legacy CAN protocol.

### 3.1. Overview of CAN with Origin Authentication and Non-Repudiation for Cyber-Attack Detection

To achieve the above factors, we define nodes that use the same secret key, limit the duration of their use of the secret key to increase security, and regulate explicit behavior for key synchronization. In this regard, CANon performs (1) grouping based on one-to-multiple communication, (2) session management different from each group, and (3) arbitrary key selection from the sequential hash chain with implicit key synchronization.

First, in CANon, according to the nature of the one-to-multiple communication of CAN, two or more nodes belong to a certain group using the same secret key, which is referred to as sender-centric group (SCG). The number of nodes in the specific group depends on the relationship between a sending node and a receiving node. For the sake of practicability in CANon, a specific SCG is designed using the information of CAN-DB. To perform origin authentication efficiently, a sending node of CANon attaches an authentication tag, which is generated by a given hash function and a selected secret key, to each message to be transmitted within a given SCG. By using the same secret key as that of the sending node, every receiving node in the given SCG participates in origin authentication and is responsible for determining whether the received message is transmitted from a legitimate sending node or not. Since every message to be shared in the specific SCG is verified for the sending node as origin authentication, the origin cannot deny the fact of its transmission. If the receiving node determines that a given message is not transmitted by the legitimate node, the message is detected as a cyber-attack and is excluded from the vehicle controls at the receiving node.

Second, CANon determines the duration of the use of the same secret key. A secret key only valid to the given SCG is never used again for origin authentication. This secret key for each transmission in the given SCG is called a one-time local authentication key (OLAK). The OLAK is replaced with a new secret key between the sending and receiving nodes without any additional synchronization process after it is consumed at the transmission of the sender and the verification of the receiver. Note that, in CANon, although the secret key given to each transmission is used as a symmetric key, any node in a given SCG does not exchange the secret key used for origin authentication in advance. Meanwhile, due to the absence of any reserved field in the CAN frame with the fixed frame length, the space available for origin authentication is very limited. While CANon inserts a hashing-based authentication tag into the CAN frame, its length is bound to a small fraction of the data field. Therefore, the relatively short length of the authentication tag makes it easy for an adversary to infer security information used for origin authentication. Moreover, if an adversary attempts numerous cyber-attacks (i.e., brute-force attacks) over a long period of time, its attacks are more likely to succeed even though the secret key is not exposed. Therefore, in order to increase the probability of neutralizing previous attempts of brute-force attacks, CANon defines a session and its duration to constrain the amount of time to ensure that the same secret key is not used again. The session start is set differently for each group except for the first session in CANon.

Third, CANon distributes a different random number, which is referred to as salt, for each SCG in order to start a new session by an enclosed key distribution and management (E-KDM) mechanism. A new salt is used for session key generation in all nodes of a given SCG so that it avoids the exposure of a session key. Furthermore, the nodes belonging to a specific SCG randomly choose one OLAK among a series of secret keys in the given SCG. When performing the arbitrary choice of OLAK, a sending node uses a random number, which is referred to as a nonce, and then informs it of the SCG by implicit key synchronization. Therefore, it reduces the key exposure occurrence much more.

In CANon, there are two types of nodes: A gateway node and a normal node. The gateway node is a CAN node connecting to another local CAN or other in-vehicle networks in order to transmit internal data to external local networks and vice versa. In CANon, a gateway node plays a major role in managing many SCGs and managing a series of individual sessions for those SCGs. To do this, a gateway node grasps CAN-DB of specific information of all messages transmitted over each local network. It continuously provides different salts for generating session keys, which are used to generate a new sequential hash chain for a given SCG. The normal nodes belong to a specific SCG and individually participate in a symmetric key-based origin authentication. One node belonging to the given SCG does not need to know the existence of the other nodes. This indicates that they do not interact with each other for origin authentication within the designated SCG. In CANon, it is assumed that the CAN nodes belonging to a given group have already one initialization vector (IV) before any manufacturer releases the vehicles. This can be performed at the vehicle manufacturing stage, whereby the IV is distributed within all nodes through a dedicated secure channel. This means that all nodes on the CAN bus have the same IV that is not used as a secret key. CANon also assumes that an adversary does not acquire any knowledge of the hash function used in key generation as mentioned in Section 2.4. The adversary is not able to access the physical memory section of CAN nodes to obtain a pre-shared IV.

### 3.2. Group Organization and Identification

To organize a sender-centric group, CANon utilizes the characteristic of one-to-many communication of CAN. In the case of CANon, normal nodes that do not belong to a certain SCG do not exist. One SCG consists of one sending node transmitting its message and one or more receiving nodes interested in that. It indicates that an SCG consists of at least two member nodes. In addition, there can be as many SCGs as the total number of messages transmitted by all sending nodes in one local network. A sending node can be designed to transmit various messages with different IDs. One receiving node may be interested in different messages from various sending nodes in order to control driving in a timely manner. This allows one receiving node to belong to many SCGs at the same time.

**Figure 2:**
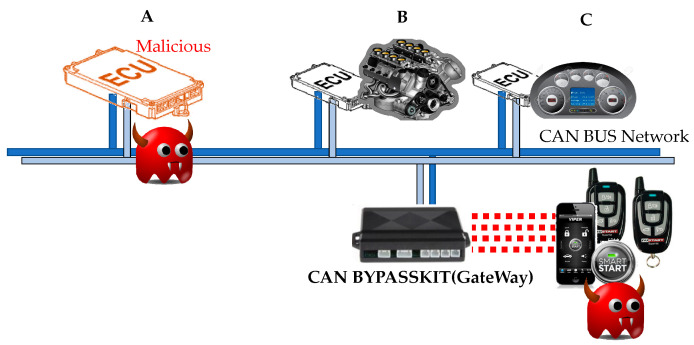
An example of CAN networks.

Figure 2 shows an example of the CAN network consisting of one gateway and three CAN nodes (i.e., ECUs). For instance, it is assumed that there are only three messages to be transmitted by three nodes in Figure 2. Among them, nodes B and C are designed as sending nodes and node A is interested in each message of the B and C nodes. However, nodes B and C are not interested in each other’s messages. Therefore, there are three SCGs: Node A belongs to the SCG for one message of sending node B, the SCG with the other message of sending node B, and the SCG for one message of sending node C at the same time. In CANon, since the SCGs are generated naturally according to the designed messages, no additional process is required for group organization. The SCGs can be identified by the identification field of a CAN message, which is defined as group identification (GID).

### 3.3. A CANon Platform

To detect cyber-attacks, a CANon platform performs two phases sequentially for each transmission under the combination of distributed origin authentication and centralized session management. First, at each node, transmission time interval-based cyber-attack detection (TTI-AD) is performed to detect replay attacks and brute-force attacks including modification attacks. Secondly, sequential hash chain-based cyber-attack detection (SHC-AD) is conducted to detect brute-force attacks including modification and masquerade attacks and replay attacks that are not detected in TTI-AD. While carrying out two phases, the E-KDM simultaneously updates a salt for each SCG under the initiative of a gateway node.

#### 3.3.1. Transmission Time Interval-Based Cyber-Attack Detection

CANon exploits the time-triggered transmissions of a sending node on the CAN bus to detect a cyber-attack. Instead of transmitting sporadic messages in real-time and safety-critical applications, all CAN frames are designed primarily to respond to unexpected situations with shorter transmission time intervals to ensure reliability [45]. Therefore, the TTI-AD phase aims to distinguish the transmissions that deviate from the designed TTI. In detail, the receiving nodes of a given SCG identify irregular transmissions inconsistent with the schedules of the CAN frame transmissions. Based on the designated CAN message transmissions, if a receiving node detects an unscheduled transmission, that transmission can be considered either a replay attack or a brute-force attack, including a modification attack. Note that we do not aim to identify the certain type of attacks injected.

**Figure 3:**
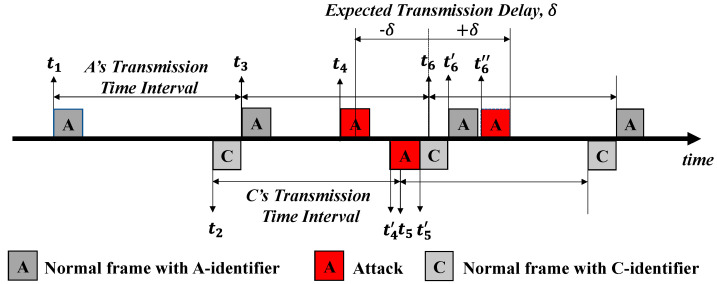
A transmission time interval-based cyber-attack detection method and the existing CAN’s problem.

For instance, there are two normal messages with the given IDs of A and C as shown in Figure 3. Each message is transmitted with its own TTI. The message with the ID of A has a higher priority than the message with the ID of C. In the case of the message with the ID of A, it has continuous intervals specified by time $t_{1}$, time $t_{3}$, and time $t_{6}$. After an adversary overhears all messages over the CAN bus, two cyber-attacks (marked in red) based on the message with the ID of A can be launched at time $t_{4}$ and time $t_{4}^{\prime }$, respectively. A receiving node detects the two messages transmitted at time $t_{4}$ and $t_{4}^{\prime }$ as cyber-attacks since the reception of the next message deviates from the expected time (i.e., time $t_{6}$) of the message with the ID of A.

Note that periodic messages are essentially transmitted by avoiding access collisions and transmission delays in the arbitration mechanism of the CAN protocol. However, an increase in a CAN bus load rate might lead to consecutive and simultaneous transmissions, which cause collisions and delays [46,47]. Meanwhile, if a large number of sporadic messages transmitted over the CAN bus suddenly occur, the transmission of the scheduled messages that are required to be delivered in time is delayed. It is possible that this delay occurs even in the case that retransmissions increase due to environmental uncertainty. When an adversary exploits such a delay to inject cyber-attacks, a receiving node takes two or more messages with the same ID in the scheduled TTI. As shown in Figure 3, in the case of the message with the ID of C, it has continuous intervals specified by time $t_{2}$ and time $t_{5}$. It is seen that the adversary’s message with the ID of A at time $t_{4}^{\prime }$ has a higher priority in transmission than the message with the ID of C at the pre-determined time $t_{5}^{}$. It is indicated that the transmission of the normal message with the ID of C at time $t_{5}$ becomes delayed by $t_{5}^{\prime }-t_{5}$. This delay leads to a consecutive delay in the transmission of the new normal message with the ID of A, which shall be scheduled at time $t_{6}$. The adversary also tries to inject a new message with the ID of A as a cyber-attack into the CAN bus at time $t_{6}^{\prime \prime }$. A receiving node takes two delayed messages with the same ID at $t_{6}^{\prime }$ and $t_{6}^{\prime \prime }$.

In the TTI-AD phase, rather than distinguishing two delayed messages with the same ID received at time $t_{6}^{\prime }$ and $t_{6}^{\prime \prime }$, CANon intends to first pick out only those messages that arrived within an acceptable period from the transmitted messages. Although it has a certain transmission delay, it accepts the message as a candidate message for the next phase to distinguish normal messages from cyber-attacks. To define the acceptable period for the candidate message, CANon uses an expected transmission delay that indicates the amount of time delayed compared to the scheduled transmission time. The expected transmission delay, denoted as δ, can be defined as follows:

(1) $$\delta =\underset{1\leq i\leq n}{\max}\left\{0,\;\mu_{i}\right\},$$

where i and n are the identifier and number of messages transmitted over the CAN bus, respectively, and $\mu_{i}$ is the maximum transmission delay time of the messages with the ID of i. In the case of Figure 3, consequently, the three messages at time $t_{4}^{\prime },\;t_{6}^{\prime },\;$and $t_{6}^{\prime \prime }$ need to be identified during the second SHC-AD phase since they exist within δ in the first TTI-AD phase. The value of δ is set to 5.41 ms by using the jitter measured from our experiment, which we discuss in Section 5.

#### 3.3.2. Sequential Hash Chain-Based Cyber-Attack Detection

In the automotive CPS, it is very important to satisfy the real-time and timeliness requirements, especially in terms of CAN frame transmission for ensuring driving safety. In this regard, CANon needs verification of the corresponding authentication tag to be completed within a predetermined TTI for each message. In CAN, since the TTI for each message can vary from five to hundreds of milliseconds depending on ECUs’ roles, in the worst case, the verification should be finished within 5 ms [14,16]. Due to its light execution, a hash function is suitable for origin authentication as it satisfies those requirements and ensures a high level of security. CANon uses a hash function to generate the authentication tags and secret keys. In CANon, the generated authentication tag is denoted by a keyed-message authentication code (KMAC). Each node uses only the contents (i.e., control data) of the data field of the CAN frame to generate KMAC and then adds it to the data field of the CAN frame for each message.

A common method to generate authentication tags is to use a one-way hash function [25,36]. It has a particular process to consume the chained hash values in the opposite direction from the generation direction after generating a hash chain with n hash values. Due to this unique feature, it satisfies the one-way property, and an adversary will find it difficult to infer the hash key used. However, in practice, there is a critical limitation of needing to generate a hash chain with a certain length n beforehand in resource-limited nodes of CAN. In other words, if n values of a hash chain are all consumed, a new chain has to be generated with a new initial secret key. At the time, generating a new hash chain may cause delayed transmissions and requires a key distribution mechanism of a new initial key. Therefore, the performance of the one-way hash function operating in CAN is closely affected by a hash table’s length. We should consider a new hash chain structure irrespective of the length of the hash chain, which does not suffer from key depletion.

**Figure 4:**
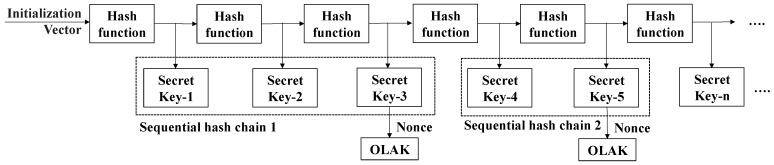
Structure of the designed sequential hash chain and selected authentication keys.

To produce KMAC, all nodes in a given SCG use an OLAK, which is selected from a sequential hash chain (SHC). The SCH is a data structure with a series of secret keys generated by the iterated hash function using the pre-shared IV as an input parameter. The security strength in CANon lies in properly determining an OLAK from the SHC generated using a given hash function. CANon introduces an element of randomness into the selection of a specific secret key as OLAK. Figure 4 presents the overview of structuring the proposed SHC by performing a given hash function at the first session and the arbitrary selection of one OLAK. When selecting an OLAK, it is randomly indexed by a given nonce in the SHC as shown in Figure 4. Since CANon can continue to generate a new secret key whenever required without depleting the keys of SHC from a given IV, it eliminates the need for CANon to store all keys of the SHC in the ECU’s memory. Whenever the OLAK is requested, the next series for SHC is generated. The OLAK selected to generate an identified KMAC for each message is never used again for a corresponding session. During a given session, the nodes in the SCG can generate an SHC with a maximum of m secret keys. The maximum length of the SHC may depend on the time taken to perform the hash function m times within the TTI of a given message. Furthermore, as a pair of sending and receiving nodes in a specific SCG uses the same OLAK selected from SHC, receiving nodes identify a suspicious frame transmission as a cyber-attack in the second SHC-AD phase and drop the corresponding message.

In the case of automotive CPS, the contents of the message sent periodically are almost similar since the message of CAN is designed to be continuously transmitted for high reliability of control, with the same control data as mentioned above. However, in CANon, due to the different OLAKs that vary for each transmission, a different KMAC is generated regardless of the content of the control data. During a given session, using a nonce to determine an OLAK randomly provides the one-way property for our CANon. There is a feature to avoid exposing the secret key wherein performing the forward selection of a secret key in SHC in such a way allows the sending node to determine a new nonce for each request and perform implicit synchronization with the receiving node. Furthermore, the security of CANon depends on the property of collision resistance of the hash function, which we discuss in Section 4.

#### 3.3.3. Enclosed-Key Distribution and Management for Session Management

If the same IV is used for a long period and the length of the secret key is short, the possibility that the same KMAC is generated increases. Since the data of the periodic messages designed for control are hardly changed during traveling except for the driver intention, it can generate the same KMACs via hash collisions. Moreover, it is possible to recover the secret key used by using a key enumeration algorithm if an adversary tries to spend too much time overhearing the messages and the KMAC over the CAN bus in order to attempt any type of cyber-attack [48].

CANon defines a session that specifies a time interval to be valid for a certain IV for SHC generation. The start of each session means a change in the existing SHC. Since a gateway node has the responsibility of managing each individual session for each SCG, it knows the start of the first transmission for each SCG. For this reason, the start time of the session for each SCG can be varied by the gateway node. To instruct the beginning of a new session, the gateway node transmits a salt to a given SCG. The i-th session key generated from the salt and GID is used as the value of the IV (denoted as $IV_{i}\;$) for the hash function. As mentioned above, the vehicle already has the pre-shared IV (denoted as Θ). To start a new session, the first session key is given as Θ of a given vehicle, namely $IV_{1}=\Theta$. With this session key, CANon generates a series of secret keys from a hash function. From the second session, the session key for each SCG is generated differently due to the different salt for each SCG and the different GID of the given SCG. At that time, the transmitted salt is implicitly used to update the existing $IV_{i}$ of the given SCG so that a new session key is never exposed for synchronizing keys.

The start of a new session for each SCG is initiated by a certain message from the gateway node, which is defined as a session expiration message (SEM). We design an SEM with the ID of the smallest value so that its transmission should be prioritized over any message on the CAN bus. Whenever the session is changed, a new SHC is re-constructed in all nodes due to a change in the session key for a new session. Sending the SEM aims to only provide a salt for each SCG to generate the next session key. All nodes of a given SCG perform the exclusive-OR (XOR) operation of their own GID and the received salt after receiving the SEM.

**Figure 5:**
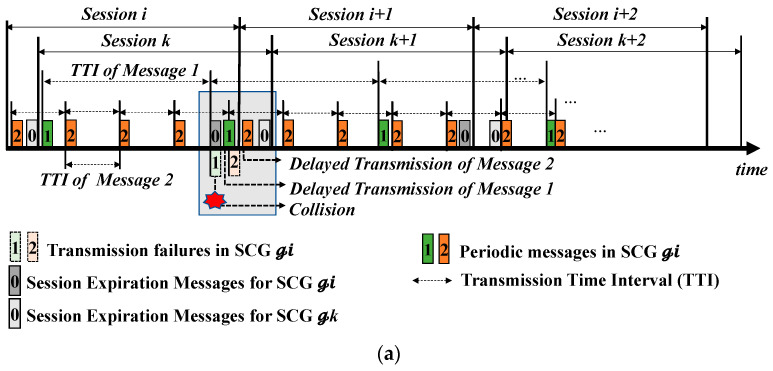

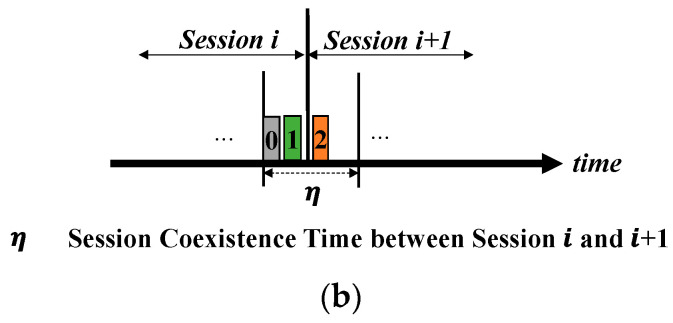
A grace period to use the old and new session keys together for origin authentication: (**a**) Problems of collisions and delayed transmissions; (**b**) session coexistence time.

Since the SEM’s transmission of a gateway node is based on event-triggered communication, the SEMs may collide with the scheduled transmission in time. Therefore, a periodic control message with a lower priority is delayed and retransmitted via the arbitration mechanism of the CAN protocol compared to the event-triggered message of the SEM with a higher priority. Such a delayed message should use the old session key during SHC-AD even if a new session key of each group is changed. Figure 5a shows a simple example of such a situation. A message, presented as an ash-colored rectangle, is the SEM of the gateway node, and the periodic messages sent by CAN nodes are presented in different colors. The smaller number in the rectangles representing the periodic messages indicates a higher priority in transmission. In the i-th session of a given SCG, the message with a lower priority of 1 collides with a higher priority of the SEM for generating the i+1th session key and the gateway node wins at this transmission. The collided message with a priority of 1 tries to be transmitted again when this CAN bus is idle. The scheduled message with a priority of 2 is impeded by this transmission. At the same time, the gateway node and the SCG first change the old session key into a new session key due to the success of SEM transmission. The delayed message with a priority of 2 is delivered after the new session starts. Although this delayed message is a normal message, it cannot be authenticated due to a new session key.

Therefore, it is necessary to provide a grace period for delayed transmissions, which allows receiving nodes to verify the KMAC using the old session key during SHC-AD. As shown in Figure 5b, to use the old session key together with the new session key, we define a session coexistence time as a grace period, denoted as η, as follows:

(2) $$\eta =\underset{n\in S}{\max}\left\{0,\tau_{trans}\left(n\right)\right\}+\tau_{auth}+\epsilon ,$$

where n is the identifier of transmitted CAN messages when S is a set of all messages to be transmitted over the CAN bus. $\tau_{trans}$ is the maximum transmission delay time measured when the periodic control messages are transmitted under the condition where there is no event-triggered message of the gateway node. $\tau_{auth}$ is the maximum time taken to authenticate a message at each node in SHC-AD. Since ϵ is an additive interval to compensate for the loss of the transmitted message in the current session, ϵ. is set as the larger value, more sufficient than the mean transmission delay.

Considering its practicability, in this paper, we determine a variable set of $\tau_{trans}$ by examining the measured jitter and $\tau_{auth}$ by examining the time required to perform authentication in real-time. In the case of $\tau_{auth}$, it is necessary to analyze the worst case in the designed CAN-DB. Since the jitter and time limit can vary depending on the CAN-DB of released vehicles, the values of the variables defined in (2) are set to be values identified by the experimental results of Section 5. Consequently, $\tau_{auth}\;$is set to 0.3974 ms and $\tau_{trans}\;$is set to 5.41 ms.

To determine the start time of the session for each SCG, a gateway node exploits the session duration, regulated by the maximum number of messages that the sending node can transmit. The session duration time for one session of a given SCG is set to 1.2 s according to the security analysis of the probability of success of the cyber-attack in Section 5.2. A gateway node transmits each SEM immediately after the time elapsed by the session duration from the time of transmitting its first message of the sending node of a given SCG.

### 3.4. CANon Operation

In this subsection, we introduce node operation according to the roles of the nodes in CANon and the detection method in detail. For that, the notations and their descriptions are shown in Table 1. The following terminology is used to effectively introduce the proposed CANon.

In CANon, a gateway node manages a series of sessions with a given time interval for each SCG as mentioned above. For example, in a given i-th session, the data field of the k-th message sent by a sending node uS is denoted as $M_{uS}^{i_{k}}$, where the sending node’s identifier is the same as the ID of its message to be transmitted over the CAN bus. A session key for each SCG is denoted as $SK_{ℊ}^{i}$, where i is used as the identifier of the *i*-th session and ℊ indicates the group identification. ℊ can be also identified by using the contents of the ID field in the message of the sending node that is only one in the given SCG. Hence, an initial session key for the given SCG ℊ in the first session is given as $SK_{ℊ}^{1}$ and is the same as the pre-shared IV Θ only at the initial state. Except for the first session, the gateway node periodically transmits SEMs and is identified by the contents of the ID field in its SEM.

#### 3.4.1. Sender Operation

For each transmission, the sender uS generates the KMAC ${\mathbb{C}}_{uS}$ using an OLAK $\Lambda_{uS}^{i_{k}}$ derived from its SHC by its nonce $nU_{}^{i_{k}}$. In the given group, to transmit its k-th message, the sender uS performs the following processes during the i-th session. First, the sender determines its own nonce $nU_{}^{i_{k}}$ representing the number of the iterations of the given hash function. Its iterated executions result in an OLAK $\Lambda_{uS}^{i_{k}}$. In other words, the latest value of its SHC becomes the OLAK. The sender’s nonce $nU_{}^{i_{k}}$ is randomly selected within a particular range between the minimum value, denoted as $\varepsilon_{min}$, and the maximum value of the iterations of the given hash function, denoted as $\varepsilon_{max}$. It is important to provide an OLAK $\Lambda_{uS}^{i_{k}}$ different from the initial session key $SK_{ℊ}^{1}$ for the given group. In CANon, the value of $\varepsilon_{min\;}$is set to 1. Since the value of $\varepsilon_{max}$ depends on the CAN node’s performance, in this paper, we intend to determine the maximum number of iterations by using the experimental results in Section 5. In detail, the maximum value is set to the number of iterations of the given hash function that can be performed sufficiently within the smallest TTI for every message sent over the CAN bus. From the experimental results, $\varepsilon_{max}$ is set to be six.

Second, after selecting nonce $nU_{}^{i_{k}}$ randomly, the given H(·) iterates nonce $nU_{}^{i_{k}}$ times to build the OLAK $\Lambda_{uS}^{i_{k}}$ of the SCH for the k-th message. First, if this is the first time in the given session, it performs the nonce $nU_{}^{i_{k}}$ times the given $H\left(\cdot \right)$ from the current session key $SK_{ℊ}^{i}$. If this is not the first time, the sender performs the nonce $nU_{}^{i_{k}}$ times the given $H\left(\cdot \right)$ from the latest OLAK $\Lambda_{uR}^{i_{k-1}}$ used previously. In addition, only if the current session i is the first session, the session key $SK_{ℊ}^{i}$ is given by Θ. Otherwise, $SK_{ℊ}^{i}$ is changed according to the E-KDM for a gateway-initiated session. Using the control data Δ, the new OLAK $\Lambda_{uS}^{i_{k}}$, and the nonce $nU_{}^{i_{k}}$, the sender uS generates its own KMAC (i.e., ${\mathbb{C}}_{uS}=H(\Lambda_{uS}^{i_{k}}\left|\left|nU_{}^{i_{k}}\right|\right|\;\Delta$)) and then transmits $M_{uS}^{i_{k}}=\left[\Delta \left|\left|nU_{}^{i_{k}}\;\right|\right|{\mathbb{C}}_{uS}\right]$.

#### 3.4.2. Receiver Operation

One or more receivers in a given SCG take the message broadcasted from the sender. Note that there is only one sender in the given SCG. They independently verify the received message. As mentioned above, if the received $M_{uS}^{i_{k}}$ does not observe the scheduled TTI, it is discarded during the TTI-AD phase.

During the SHC-AD phase, the receiver performs as follows. First, in a given SCG, the receiver uR extracts the data (i.e., $M_{uS}^{i_{k}}=\left[\Delta \left|\left|nU_{}^{i_{k}}\right|\right|{\mathbb{C}}_{uS}\right]$) of the data field from the CAN frame received from the sender. By using the sender’s nonce $nU_{}^{i_{k}}$, the receiver synchronizes its own SHC with the sender’s. This synchronization indicates generating the same OLAK by performing the iteration of the given hash function. Therefore, it implicitly synchronizes its own SHC with the sender’s SHC using the received nonce $nU_{}^{i_{k}}\;$of $M_{uS}^{i_{k}}$. If this synchronization is the first iteration in the given session, it performs the nonce $nU_{}^{i_{k}}$ times the given $H\left(\cdot \right)$ from the current session key $SK_{ℊ}^{i}$. If this synchronization is not the first time, the receiver performs the nonce $nU_{}^{i_{k}}$ times the given $H\left(\cdot \right)$ from the latest OLAK $\Lambda_{uR}^{i_{k-1}}$ used previously. The iterated operations result in building its SHC with a length of $nU_{}^{i_{k}}$. The latest value of this constructed SCH becomes the receiver’s OLAK $\Lambda_{uR}^{i_{k}}$ that is the same as the sender’s OLAK $\Lambda_{uS}^{i_{k}},\;$which is used to generate a receiver’s KMAC ${\mathbb{C}}_{uR}$. At that time, all nodes of the given SCG use the same session key $SK_{ℊ}^{i}$ for the SHC during the i session. If i=1, $SK_{ℊ}^{i}=\Theta$ is used as the first input value to $H\left(\cdot \right)$. Otherwise, $SK_{ℊ}^{i}$ is changed periodically according to the E-KDM. Second, the receiver uR compares its own KMAC ${\mathbb{C}}_{uR}$ with the sender’s KMAC ${\mathbb{C}}_{uS}$ to verify the legitimate message of the sender uS. If both values are not matched, $M_{uS}^{i_{k}}$ should be discarded since it is determined as a cyber-attack. We provide Algorithm 1 as the sender operation described in Section 3.4.2 and Algorithm 2 as the receiver operation.
**Algorithm 1: Sender Operations**Input:Known $SK_{G}^{i}$, Δ, i, k, and uS Given n=1 Given $\varepsilon_{max}$, the maximum number of iterations of a hash functionOutput:$M_{uS}^{i_{k}}=\left[\Delta \left|\left|nU_{}^{i_{k}}\right|\right|{\mathbb{C}}_{uS}\right]$1$nU_{}^{i_{k}}\;$= an integer value between 1 and $\varepsilon_{max}$2**While**n ≤ $nU_{}^{i_{k}}\;$3**If**n = 1 **Then**4χ = $H\left(SK_{ℊ}^{i}\right)$5   **Else**6     χ = $H\left(\chi \right)$7   **End If**8**End While**9$\Lambda_{uS}^{i_{k}}$ = χ10${\mathbb{C}}_{uS}$ = $H\left(\Lambda_{uS}^{i_{k}}\left|\left|nU_{}^{i_{k}}\right|\right|\;\Delta \right)$11$M_{uS}^{i_{k}}=\left[\Delta \left|\left|nU_{}^{i_{k}}\right|\right|{\mathbb{C}}_{uS}\right]$12**Transmit**$M_{uS}^{i_{k}}$


**Algorithm 2: Receiver Operations during SHC-AD**
Input:Known $SK_{ℊ}^{i}$, $M_{uS}^{i_{k}},\;\;i$, k, and uR Given n=1Output:Boolean1Extract Δ  from $M_{uS}^{i_{k}}=\left[\Delta \left|\left|nU_{}^{i_{k}}\right|\right|{\mathbb{C}}_{uS}\right]$2Extract $nU_{}^{i_{k}}$ from $M_{uS}^{i_{k}}=\left[\Delta \left|\left|nU_{}^{i_{k}}\right|\right|{\mathbb{C}}_{uS}\right]$3Extract ${\mathbb{C}}_{uS}$ from $M_{uS}^{i_{k}}=\left[\Delta \left|\left|nU_{}^{i_{k}}\right|\right|{\mathbb{C}}_{uS}\right]$4**While**n ≤ $nU_{}^{i_{k}}$5   **If**
n = 1 **Then**6χ  = $H\left(SK_{ℊ}^{i}\right)$7   **Else**8     χ  = $H\left(\chi \right)$9   **End If**10
**End While**
11$\Lambda_{uR}^{i_{k}}$ = χ 12${\mathbb{C}}_{uR}$ = $H\left(\Lambda_{uR}^{i_{k}}\left|\left|nU_{}^{i_{k}}\right|\right|\;\Delta \right)$13**IF**${\mathbb{C}}_{uR}$ = ${\mathbb{C}}_{uS}$
**Then**14   Verify ${\mathbb{C}}_{uS}$ as **TRUE**15
**Else**
16   Verify ${\mathbb{C}}_{uS}$ as **FALSE**17Discard $M_{uS}^{i_{k}}$18
**End If**


#### 3.4.3. Gateway Operation

In CANon, a new session indicates that a given SCG should obtain a new session key for building its new SHC. The start of a new session for each SCG is designated by a gateway node. The processes of updating each session are the same as Algorithm 1 of the sender and Algorithm 2 of the receiver for the SHC-AD phase except for the process of determining the number of iterations of $H\left(\cdot \right)$ to generate a new session key.

In updating the current session, the gateway node and all nodes of the given SCG participate. The gateway uG sequentially transmits SEMs for each group with a predetermined session duration time. Every session after the first session becomes a gateway-initiated session. A gateway-initiated session for each group is defined as being initiated when the gateway uG transmits the SEM (i.e, $SEM_{uG}^{i}=\left[\Delta \|nG^{i}\|{\mathbb{C}}_{uG}\right]).$ As mentioned above, the SEM is defined as an event-triggered message with the highest priority compared to any other periodic message. To generate this SEM, a gateway node randomly selects the salt $nG^{i}$. The minimum and maximum values of the salt $nG^{i}$ are determined in the same way as the nonce $nU_{}^{i_{k}}$ described in Section 3.4.2.

To prove a gateway node itself, it provides its KMAC by performing a given $H\left(\cdot \right)$ $nG^{i}$ times from the existing session key $SK_{ℊ}^{i}$ for SCG $ℊ\;\left(only\;if\;i=0,\;SK_{ℊ}^{i}=\Theta \right)$. By using three values of the session key $SK_{ℊ}^{i}$, the salt $nG^{i}$, and the control data Δ that is padded with randomized bits, the gateway node generates its KMAC (i.e., ${\mathbb{C}}_{uG}$ = $H\left(SK_{ℊ}^{i}\left|\left|nG^{i}\right|\right|\;\Delta \right))$ based on the given $H\left(\cdot \right)$. After the gateway node transmits its message $SEM_{uG}^{i_{}}=\left[\Delta \left|\left|nG^{i}\right|\right|{\mathbb{C}}_{uG}\right]$, all nodes in the given SCG ℊ verify the KMAC of the origin and then generate a new session key $SK_{ℊ}^{i+1}$. The next session key $SK_{ℊ}^{i+1}$ for each SCG also needs to be stored in the gateway node in order to perform the session synchronization between the gateway node and the SCGs. Both the salt $nG^{i}\;$and GID ℊ are used to generate the next session key, which is used to create a series of secret keys.

The gateway node performs $\mathrm{RShift}\left(\cdot \right)$ after conducting the bitwise-exclusive OR operation (i.e., $ℊ⊕nG^{i}$) so that a new nonce lies in the range of $\varepsilon_{min}$ to $\varepsilon_{max}$. The $\mathrm{RShift}\left(\cdot \right)$ function fills each bit with zeros from the most-significant bit to the least-significant bit until the result of $ℊ⊕nG^{i}$ is less than or equal to $\varepsilon_{max}$. Therefore, it aims to generate as many executions of $H\left(\cdot \right)$ as possible within a given time on a resource-limited normal node. When updating the current session, the gateway node performs the process presented in Algorithm 3.

The SEM message of the gateway node instructs a specific SCG ℊ to start a new session by replacing the old session key with the new session key. After all of the SCG verifies the SEM of the gateway node, to generate a new session key $SK_{ℊ}^{i+1}$ for the next session i+1, all of the nodes repeatedly perform the given hash operation with the current session key $SK_{ℊ}^{i}$. The number of iterations of the given $H\left(\cdot \right)$ is determined by the result of $\mathrm{RShift}\left(G⊕nG_{}^{i+1}\right)$. Since each SCG has a different identifier, the new session key $SK_{ℊ}^{i+1}\;$is determined differently on each SCG. We present the operation of the SCG starting a gateway-initiated session in Algorithm 4.
**Algorithm 3: Gateway Operations in E-KDM**Input:Known $SK_{ℊ}^{i}$, i, and uG Given n=1, Δ = bits padded with randomized bits, k = 0Output:$SK_{ℊ}^{i+1}$, $SEM_{uG}^{i+1_{}}$1$nG_{}^{i+1}\;$= an integer value between 1 and $\varepsilon_{max}$2${\mathbb{C}}_{uG}$ = $H\left(SK_{ℊ}^{i}\left|\left|nG_{}^{i+1}\right|\right|\;\Delta \right)$3$SEM_{uG}^{i+1_{}}=\left[\Delta \left|\left|nG_{}^{i+1}\right|\right|{\mathbb{C}}_{uG}\right]$4ε**=**$RShift\left(G⊕nG_{}^{i+1}\right)$5χ = $SK_{ℊ}^{i}$6**While**n ≤ ε7   χ = $H\left(\chi \right)$8**End While**9$SK_{ℊ}^{i+1}$ = χ10**Transmit**$SEM_{uG}^{i+1_{}}$11**Return**$\;SK_{ℊ}^{i+1}$


**Algorithm 4: Group Operations in E-KDM**
Input:Known $SK_{ℊ}^{i}$, $SEM_{uG}^{i+1}$, ℊ, and i Given n=1Output:

$SK_{ℊ}^{i+1}$

1Extract Δ from $SEM_{uG}^{i+1}=\left[\Delta \left|\left|nG_{}^{i+1}\right|\right|{\mathbb{C}}_{uG}\right]$2Extract $nG_{}^{i+1}$ from $SEM_{uG}^{i+1}=\left[\Delta \left|\left|nG_{}^{i+1}\right|\right|{\mathbb{C}}_{uG}\right]$3Extract ${\mathbb{C}}_{uG}\;$from $SEM_{uG}^{i+1}=\left[\Delta \left|\left|nG_{}^{i+1}\right|\right|{\mathbb{C}}_{uG}\right]$4${\mathbb{C}}_{uR}$ = $H\left(SK_{ℊ}^{i}\left|\left|nG^{i}\right|\right|\;\Delta \right)$5**IF**${\mathbb{C}}_{uR}$ == ${\mathbb{C}}_{uG}$
**Then**6   Verify ${\mathbb{C}}_{uG}$ as **TRUE**7
**Else**
8   Verify ${\mathbb{C}}_{uG}$ as **FALSE**9   Discard $SEM_{uG}^{i+1_{}}$10   **Return**11
**End If**
12

ε

**=**

$RShift\left(ℊ⊕nG_{}^{i+1}\right)$

13χ = $SK_{ℊ}^{i}$14**While**n ≤ ε15   χ = $H\left(\chi \right)$16
**End While**
17$SK_{ℊ}^{i+1}$ = χ18
**Return**

$SK_{ℊ}^{i+1}$



## 4. Security Analysis

### 4.1. Probability of Success of Cyber-Attack

As mentioned above in Section 2.4, the cyber-attacks we consider in the in-vehicle network are largely divided into two categories: A replay attack and a brute-force attack. In the case of the replay attack, after the adversary stores the eavesdropping message, it re-transmits the same message with a delay over the CAN bus. At that time, the adversary does not modify the contents of the message. If the message with the falsified control data is transmitted with a delay after eavesdropping, this cyber-attack is referred to as a brute-force attack, including both modification and masquerade attacks. CANon guarantees the detection of all injected replay attacks by the receiving nodes of a given SCG since the OLAK is changed for every message.

In the case of a brute-force attack, the probability of success of the cyber-attack may depend on the length of the data field and the length of TTI. This cyber-attack aims to disrupt driving safety by allowing an adversary to succeed in any one of several attempts.

In a brute-force attack, the adversary abusing the message ID of a legitimate node injects arbitrary data into the CAN bus. This is because any injected brute-force attack is discarded at the receiving node unless the adversary captures the message ID of a legitimate node. In this regard, since the adversary could design more sophisticated attacks, it takes the form of a masquerade attack or a modification attack, and the forged or arbitrary control data can be set in the data fields of the CAN message. As mentioned in Section 2.1, the length of the CAN data field is only 8 bytes. CANon exploits this data field of the CAN frame, which consists of three parts: Control data, a nonce, and KMAC. When an adversary tries to reconstruct the contents of this data field to succeed in a brute-force attack, it should focus on the two remaining parts, excluding the falsified control data. This is because the adversary is sufficiently able to determine the fact that the control data are rarely changed from periodic transmissions of the legitimate CAN node.

In this regard, we note that the success of the brute-force attack depends on the lengths of the KMAC and the nonce. The total length of the KMAC and the nonce is available up to 8 bytes if there are no control data to be transmitted. Hence, there exists a total number of $2^{\left(64-d\right)}$ cases of brute-force attacks, where the number of bits of the control data to be transmitted is denoted as d. Considering only brute-force attacks, the success rate of those, denoted as$\;S_{\mu }$, is given as:

(3) $$S_{\mu }=\frac{1}{2^{\left(64-d\right)}}$$

In order to increase the probability of success of the cyber-attack, an adversary intends to inject the brute-force attack over the CAN bus according to a given TTI of a legitimate CAN node. The receiving nodes in the SCG are able to detect it, except in the case of determining the received message as a normal message due to the hash collision occurrence, even though that message is a cyber-attack. A hashing operation to generate KMAC may typically cause the hash collision occurrence, which produces the same hash values with different inputs. For this reason, the probability of success of the cyber-attack is equal to the hash collision occurrence rate. In addition, the possibility of the hash collision occurrence can be described by using the well-known birthday paradox that is introduced as a counterintuitive observation [49]. Based on the birthday paradox, we formulate the probability of success of the cyber-attack, which is denoted as $p\left(n;\phi \right)$. It indicates the probability of a hash collision occurring when there are n CAN messages for one session.

(4) $$p\left(n;\phi \right)=1-\frac{\phi !}{\phi^{n}\left(\phi -n\right)!},$$

where ϕ is the number of different hash values (i.e., KMACs) that can be generated within one session and n is the maximum number of CAN messages that can be generated for a given session. Note that the maximum number n of CAN messages transmitted for one session is not only the same as the length of the SCH, but it is also the same as the number of CAN messages to be manipulated by the adversary for one session. The number of hash values, denoted as ϕ, varies as a function of the length of KMAC. The data field of the CAN frame is usually composed of at least one byte of control data. In CANon, it is assumed that the maximum length for KMAC is limited to 32 bits, which is half the length of the data field. From the results of (4), Table 2 shows the probability of success of a cyber-attack as a function of the length of KMAC.

The results of Table 2 indicate that brute-force attacks hardly succeed when the length of KMAC is over 24 bits. In particular, it is shown that when the number of CAN messages is between 25 and 50, they provide very low success probabilities of 0.0017% and 0.0073%, respectively, during one session. Moreover, if the number of CAN messages is below 200 at a KMAC length of 32 bits, it is difficult for an adversary to succeed. From the results of Table 2, it is theoretically proven that CANon is capable of guaranteeing security against brute-force attacks under certain conditions of a given KMAC length and a given number of CAN messages. To validate the performance of CANon under the maximally acceptable condition from Table 2, the condition (i.e., a 32-bit KMAC and 3000 messages) with a collision probability of 0.104% is used in our experiment.

### 4.2. Analysis of Key Freshness

To detect cyber-attacks in the in-vehicle network, CANon uses two secret keys: An OLAK and a session key. In the case of the OLAK, its freshness is guaranteed by the randomness while a sending node selects it. In addition, it is valid only for the TTI of the transmitted message. That is, the OLAK of CANon is valid with a minimum of 5, which is the smallest TTI of the CAN message according to the CAN-DB.

In CANon, even when assuming that the first part of the current message to be transmitted is constructed with the same value as that (e.g., the ID field and the control field) of the previous message, the message is varied. This is because the data field is filled with the control data and the algorithmic data by CANon. However, the values of the data field in the individual messages generated from two OLAKs may be the same if a hash collision of two different OLAKs occurs when the control data between two messages is the same. To minimize these collision occurrences, CANon defines the duration time of one session as the amount of time taken for a sending node to transmit messages with different values. In other words, CANon intends to continue the low probability of hash collision occurrences during one session by using the values of Table 2. Therefore, the same bit-streams within a given session hardly exist as the same messages cannot be created twice or more, with a 99.9% probability under our experimental conditions (i.e., a 32-bit KMAC and 3000 messages).

In the case of the session key, the level of the key freshness may depend on the duration time of the session. At the start of each session, the nodes belonging to the SCG receive a newly assigned salt to generate a session key that contributes to preventing the exposure of the key. Furthermore, the session key is not used directly to transmit the message. Even if an adversary determines a certain pattern of the contents of the eavesdropping CAN message, the obtained information is only valid to be exploited for the corresponding session since it is useless in the new session. Nevertheless, to perform a cyber-attack based on the obtained session key, the adversary needs more information. Moreover, CANon regulates the start time of each session with a fixed duration time for every SCG. The freshness of the session key is achieved for the session duration time.

## 5. Performance Evaluation

In this section, we evaluate the performance of CANon under the CAN environment, which is constructed via a combination of (1) a testing software tool (i.e., CANoe) used by automotive manufacturers and ECU suppliers, (2) real ECUs mounted in automobiles for CAN, and (3) the message sets of the SAE benchmark [16,18]. We add the message of the gateway to the message sets of the SAE benchmark, as shown in Table 3.

### 5.1. Experimental Environment

**Figure 6:**
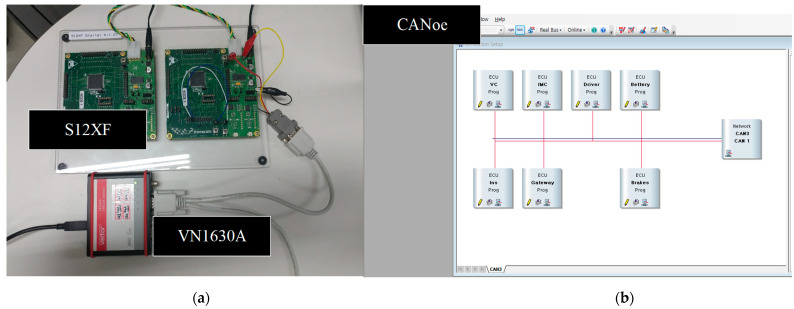
An experimental environment: (**a**) A snapshot of hardware components connected to CANoe; (**b**) a topology of the CAN network on CANoe.

We build an experimental in-vehicle network environment with a hardware ECU node using Freescale S12XF board and software ECU nodes through the CANoe testing software. The constructed environment is shown in Figure 6.

The nodes of CANon use a hash function using a dynamic linking library (DLL), and the functionality of CANon is implemented by the communication access programming language (CAPL). In our experiment, the designated in-vehicle network consists of eight CAN nodes: Seven nodes referring to the SAE benchmark and one gateway node. By using a Freescale S12XF board, one real CAN node is implemented as the transmission control. Each of the seven virtual nodes of CANoe plays the roles of battery control, vehicle controller (V/C), inverter/motor controller (I/M C), instrument panel display control, driver inputs control, brakes control, and one gateway node, respectively. Every node of the CAN network is capable of transmitting, as well as receiving, messages. Table 4 shows the specification of the tools that constructed our experimental environment.

As mentioned above, the data field of the CAN frame is defined to deliver control data with 8 bytes. To perform transmission of the message through CANon, we reconstruct the data field with the control data of 3 bytes limited to the SAE benchmark, a nonce of 1 byte, and a KMAC of 4 bytes.

### 5.2. Determination of Variables

It is important to determine the values of the variable set of $\tau_{auth}\;$and $\tau_{trans}$ denoted in Equation (2) for CANon. Determining the individual values of this variable set has the effect of determination of the session coexistence time η. $\tau_{auth}\;$of this variable set is also used to determine the session duration time for each SCG. The value of $\tau_{trans}$ is the same as that of δ of Equation (1). Moreover, in this subsection, we determine the hash function to be used for generating and verifying KMACs and the maximum length of SHC per session.

First, CANon should determine one element of the variable set, namely $\tau_{auth}$, which represents the maximum amount of time allowable to perform one-time origin authentication in real-time. $\tau_{auth}^{i}$ of the sending node i is given by:

(5) $$\tau_{auth}^{i}=\frac{t}{\left(n_{snt}+n_{recv}\right)},$$

where $n_{snt}$ and $n_{recv}$ are the numbers of sent and received messages, respectively, from node i for unit time t. From Equation (5), $\tau_{auth}$ can be derived as

(6) $$\tau_{auth}=\underset{i}{\max}\{\tau_{auth}^{i},\;0\}.$$

To set the variable $\tau_{auth}$, we investigate the total number of message transmissions that should be completed during a given period, and for this, we utilize a certain node with the maximum load in terms of message transmission. In our experimental environment based on the SAE benchmark, the V/C node performs the most transmissions of CAN messages over the CAN bus per unit time t. In detail, since the V/C node sends and receives 822 and 1684 CAN messages, respectively, for every 1 s, it takes at least 0.3974 ms for the V/C node to complete its transmission. Therefore, $\tau_{auth}$ is set to 0.3974 ms in the worst-case scenario since that of Equation (2) requires the completion of verifying KMAC.

**Figure 7:**
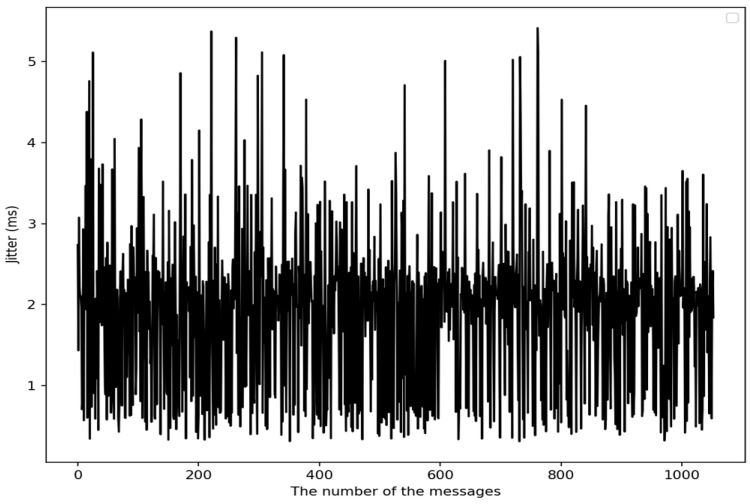
Jitter of the lowest CAN message transmitted over the CAN bus.

Second, to determine the value of the second element of the variable set, namely $\tau_{trans}$, we measure the jitter of the transmitted messages. The maximum jitter among measurements can determine the expected transmission delay δ of Equation (1). In this paper, in order to grasp the variable instinctively, the jitter is presented as the transmission delay of the CAN messages even though the term jitter typically refers to the variation in the transmission delay. Note that, in the CAN protocol, the transmission of the CAN message with the highest priority (i.e., the lowest ID) is never affected by other transmissions. Therefore, the jitter is measured for the CAN message with the highest priority presented in the SAE benchmark. Figure 7 presents the measured jitter as a function of the number of messages transmitted. As it is shown that the longest jitter representing the worst case is 5.41 ms, $\tau_{trans}$ is set to the longest jitter, which indicates the maximum transmission delay in Equation (2). From the experiment results, the session coexistence time η is set to 10 ms, which is rounded off below the decimal point of the sum of the maximum of $\tau_{trans}$, $\tau_{auth}$ (i.e., 0.3974 ms), and ϵ.

Third, to determine the session duration time, we exploit both the number of messages that can be transmitted per session and the time allowable for authentication ($\tau_{auth})$. In our environment, a size of 32 bits is assigned to KMAC. This is because, as shown in Table 2, the collision probability approximately reaches the value of 0.105%, which is acceptable when the length of KMAC (ϕ) is 32 bits and the number of messages (n) is 3000. We set the session duration time to 1.2 s which is obtained by multiplying $\tau_{auth}$ of the V/C node by n of 3000.

Fourth, we suggest a hash function suitable to generate OLAK and verify KMAC in SCG. For the CAN protocol, CANon utilizes a simple algorithm. A hash function is typically classified into a cryptographic hash function and a non-cryptographic hash function. In general, the cryptographic hash function is known as having higher security than a non-cryptographic hash function. Meanwhile, the non-cryptographic hash function has lower complexity in terms of computation than the cryptographic hash function, which requires a great deal of processing time and system resources [48,50]. In order to determine the hash function used in CANon, considering our environment, we measure the time taken to perform several hash functions to create OLAK and verify KMAC, among various hash functions that are frequently used. We perform a comparison of SHA-256, SHA-1, and the modified CRC [51,52]. The modified CRC is not a standard CRC-32 checksum and destroys the linearity as the modified CRC utilizes the IV changed for each message [51,52]. In this paper, it is already assumed that the modified CRC is a secret that is known only to the sender and receiver CAN nodes. Table 5 shows the mean time taken for executing each hash function, including SAH-256, SHA-1, and the modified CRC32, which is denoted as mCRC32, and is 1000 times in the real node. It indicated that SHA-256 and SHA-1 are not suitable for CANon as performing the given tasks takes much more time than $\tau_{auth}$ (i.e., 0.3974 ms). Hence, the modified CRC is used in our experiments due to the resource-limited CAN node. For practicability, considering that the virtual nodes of CANoe execute the modified CRC32 much faster than the real node does, we assign the specific amount of time as a delay to virtual nodes for every calculation. This leads to the synchronization of the execution time between real and virtual nodes.

**Figure 8:**
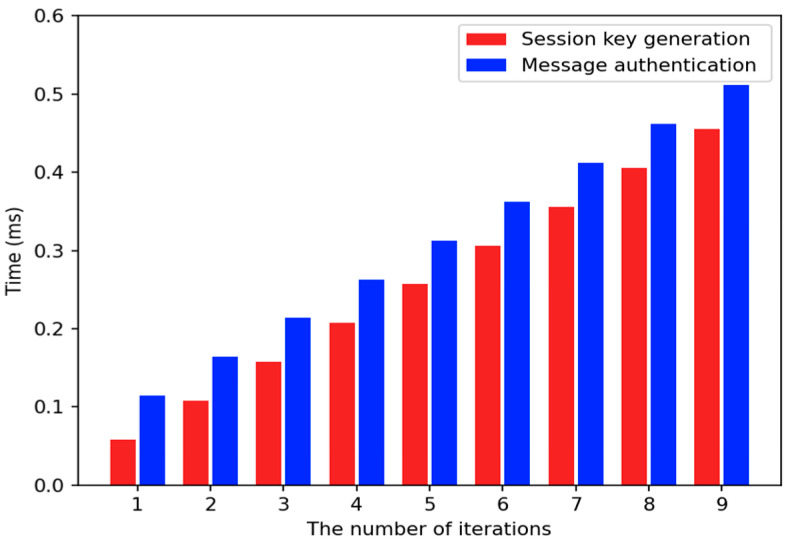
Time taken to verify the CAN message and update a gateway-initiated session as a function of iterations of a given hash function.

Lastly, we need to examine the maximum value, denoted as $\varepsilon_{max}$, of $nU_{}^{i_{k}}$, in order to define the range for $nU_{}^{i_{k}}$ (i.e., $nU_{}^{i_{k}}=[x|\;1\leq x\leq \varepsilon_{max}]$). The amount of time required for authentication should be highly affected by the number of iterations of performing the hash function, except for the amount of time taken to generate a new session key. We have already determined that the time taken for authentication for each CAN node should not exceed $\tau_{auth}$ of 0.3974 ms. Hence, the maximum number (i.e., $\varepsilon_{max}$) of iterations is bound to the given $\tau_{auth}$. Figure 8 presents the mean time taken to authenticate each CAN message and the mean time taken to generate a new session key for each session as a column chart. They are measured for 10,000 trials in the real node with an increase in the number of iterations of performing hash functions. From Figure 8, it is seen that the time taken to generate a session key is less than the time taken to authenticate the message because it additionally requires the time to generate and verify KMAC. When the number of iterations reaches six, we find that the mean time for performing authentication is 0.362547 ms, which does not exceed the value of $\tau_{auth}$. Hence, in CANon, $\varepsilon_{max}$ is set to six.

### 5.3. Experimental Results

In this subsection, we evaluate the performance of CANon in terms of the detection rate against brute-force attacks and delayed-replay attacks. All of the results are presented with an increase in the load rate of the CAN bus, which is related to the number of injected cyber-attacks (i.e., a cyber-attack rate). To inject cyber-attacks into the CAN bus, there is one virtual node acting as an adversary that is added to our experiment environment. From several experimental results, it is known that if the load rate of the CAN bus exceeds 70%, most of the transmitted messages are lost and CAN falls into a bus-off state by the generated error frames [14,18,46,47]. Therefore, in our experiment, the sum of the transmission rates of the adversary and the legitimated CAN nodes are not able to exceed 70% of the CAN bus load.

#### 5.3.1. CANon’s Defensibility

The adversary aims to compromise the control of the targeted vehicle, rather than stop its operation, so it injects manipulation under three scenarios: Only brute-force attacks occur, only delayed-replay attacks occur, and both attacks coexist. For all cyber-attacks, the TTI of the manipulated messages ranges from 5 to 10 ms.

In our experiment, a brute-force attack is conducted by the adversary node that sends its manipulated CAN message with a data field of arbitrary generated values among IDs less than the value of 6. After all the messages that violate the regular transmission schedule are first detected and then eliminated in the TTI-AD phase, the remaining CAN messages enter the SHC-AD phase. Table 6 shows the performance of detection against brute-force attacks as a function of the bus load rate. All values are rounded to the first decimal place as shown in Table 6. It is shown that when the bus load rate is 58.7%, the brute-force attack comprises 3.87% (i.e., 8554) of the total number of messages transmitted over the CAN bus. As the bus load rate increases, the detection rate against cyber-attacks increases in the first TTI-AD phase. This is because the adversary transmits its message regardless of regular transmission schedules of the legitimate messages. When the bus load rate reaches a lower value, it is likely that the adversary enables the brute-force attacks to be injected at roughly the same TTI as the regular TTI or with little collision delay. Therefore, at a lower bus load rate, many more cyber-attacks are detected in the second SHC-AD using KMAC and OLAK. Furthermore, from the results of Table 6, it is seen that CANon does not allow false-positive detection of cyber-attacks. Hence, these results demonstrate that CANon is robust against brute-force attacks.

Second, the adversary conducts the delayed-replay attack by capturing the data field of the message transmitted by a normal node. After a specific time interval, the adversary node sends its own message with the same contents of that data field. Although the TTI is randomly selected for every attack injection, Table 7 presents the performance of detection against delayed-replay attacks as a function of the bus load rate. In contrast to brute-force attacks, delayed-replay attacks cannot be frequently injected into the CAN bus. This is because such attacks can only be carried out by capturing the legitimate messages sent in the given TTI. In this regard, when the bus load rate is low, the number of injected cyber-attacks is very small. As mentioned above, the adversary randomly chooses its TTI for each transmission in the specific range of 5 ms to 10 ms. Given the TTI of this cyber-attack is 5 ms, it tends to fall within the expected transmission delay δ of 5.41 ms configurated in CANon. This makes it difficult to detect this type of cyber-attack based on the interval in the first TTI-AD phase. These results can be seen at higher bus load rates (≥61.1%) in Table 7. In the case of the lowest bus load rate in Table 7, it is seen that all cyber-attacks are detected in the first phase because they significantly deviate from the specified transmission delay.

Lastly, the adversary aims to conduct a series of brute-force and delayed-replay attacks. This experiment is conducted in a real environment where both cyber-attacks exist. The configuration for each attack is the same as those of the first and second cyber-attacks described above, respectively. The results shown in Table 8 evidence the fact that the brute-force attacks injected at a random TTI are identified effectively in the TTI-AD phase, while the delayed-replay attacks injected with legitimate contents are accurately identified in the SCH-AD phase using the OLAK and KMAC-based detection method.

In the case of the brute-force attack, our finding is that the second phase (i.e., SHC-AD) has better detection performance than that of the first phase (i.e., TTI-AD) under lower bus loads. As the bus load increases, the performance during the first phase is gradually improved. In contrast to CANon’s defensibility against brute-force attacks, in the case of delayed-replay attacks, it is found that the performance of the second phase is significantly better than that of the first phase when the bus load is increased. From these results, it is assumed that during the first phase, CANon is robust against brute-force attacks, and during the second phase, it is robust against delayed-replay attacks. Therefore, the results shown in Table 8 support our above assumptions. In a real environment where both cyber-attacks exist at the same time, CANon shows good performance, as shown in Table 8, in which the first and second phases can be used complementarily to each other under a higher bus load.

#### 5.3.2. Comparison of Theoretical Analysis and Experimental Performance

**Figure 9:**
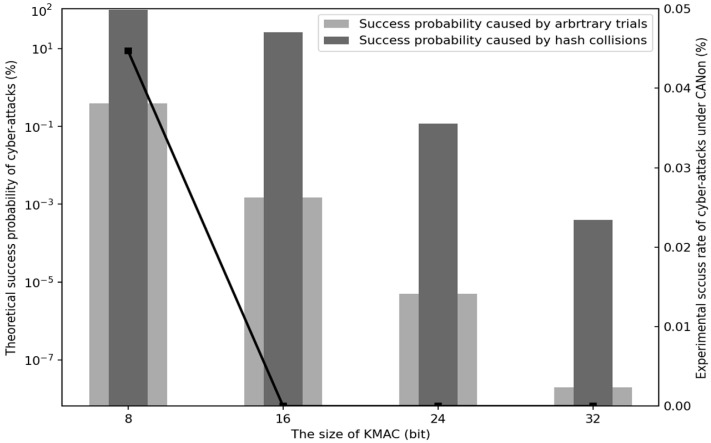
Comparison of the cyber-attack success rate between the theoretical analysis and the experiment as a function of the size of KMAC.

To make general conclusions, we compare the results of theoretical analysis with our experiments as a function of the length of KMAC. Figure 9 presents the comparison of the results between the theoretical analysis and the experiment when the bus load rate is given as 63.76%. In Figure 9, two theoretical results include the cyber-attack success probability related to the length of KMAC in Equation (3) and that related to the length of KMAC and the hash collision occurrence when the number of the messages is 200, given in Table 2. They are shown with the cyber-attack success rate of CANon under a series of brute-force and delayed-replay attacks.

As shown in Figure 9, the result of Equation (3) marked in light gray decreases as a function of the length of KMAC, and the result of Equation (4) marked in dark gray also tends to a decrease, as shown in Table 2. The result of CANon outperforms the theoretical results, regardless of the length of KMAC, since CANon is capable of identifying suspicious messages as cyber-attacks during the first phase of two phases. It is shown that in the case of the shortest-length KMAC of 8 bits, the experimental result of CANon is similar to the result of Equation (3) in the theoretical analysis. This indicates that the probability of hash collision occurrence is too relatively high under the condition that a pool of generated KMACs is very small due to the short length of KMAC. Nevertheless, note that the value of the cyber-attack success rate against CANon is only 0.0447%. The comparison result demonstrates that CANon based on two phases provides robustness and enhanced security for CAN against brute-force and replay attacks even with the 16-bit KMAC.

## 6. Conclusions

To conduct lightweight and fast detection against cyber-attacks, this paper proposed CANon, which provides a realistic and practical solution for the existing CAN platform without modification. To ensure its efficiency, CANon designs a combined approach of centralized session management and distributed group authentication. In centralized session management, a gateway node is responsible for managing designated groups and every session of each group. Group authentication is distributed to all CAN nodes except for the gateway node. For the sake of low design complexity, according to the nature of the CAN protocol, some CAN nodes are grouped around a sender node and independently participate in authenticating an origin node of its group.

To reduce key exposure occurrences derived from key re-distribution, CANon does not distribute or share any secret key over the CAN bus. Instead of key distribution, CANon is designed using a sequential hash chain only valid to a particular session and a one-time local authentication key only valid to individual transmission during the given session. To improve security, authentication keys are randomly selected, and CANon randomly assigns the new state of being to the sequential hash chain at the start of a given session for each group.

To evaluate the performance of CANon, we conducted both a theoretical security analysis and an experimental analysis. First, in the theoretical analysis, we examined the robustness of CANon against cyber-attacks in terms of hash collision probabilities as the length of the hashed key is varied. Second, in the case of the experimental analysis, an experimental environment was constructed with real CAN nodes of Freescale S12XF ECUs and virtual CAN nodes of CANoe. We evaluated the performance of CANon in terms of its defense performance against cyber-attacks and the practicality of real-time processing. It can be seen that the detection rate of CANon against brute-force and replay attacks reaches 100% when the length of KMAC is over 16 bits. These results demonstrate that CANon can provide high security even in CAN messages where the length of the data field is only 64 bits.

The experimental results demonstrate that even resource-limited CAN nodes are sufficient in detecting cyber-attacks by performing origin authentication within a given limited time while providing a high level of security for transmitted messages.

## Figures and Tables

**Table 1:** Summary of notations used for CANon.

Notation	Description	Notation	Description
$u\left[S\left|R\right|G\right]$	A CAN node that is divided into sender uS, receiver uR, and gateway uG	${\mathbb{C}}_{u\left[S\left|R\right|G\right]}$	A keyed-message authentication code (KMAC)
*G*	A sender-centric group (SCG)	χ	An output of a hash function
i	A session’s identifier	H (·)	A hash function
Θ	A pre-shared initialization vector (IV)	||	Concatenation operation
$M_{u\left[S\left|R\right|G\right]}^{i_{k}}$	The data of the data field of the k-th message transmitted by node u at the i-th session	$SEM_{uG}^{i}$	A message of the gateway uG to update an old session into a new session for i-th session
Δ	Control data of the data field in CAN frame	$nU_{}^{i_{k}}$	A normal node’s nonce to generate a local one-time authentication key
$nG^{i}$	A gateway node’s salt to generate the next session key for a given SCG	$SK_{ℊ}^{i}$	A session key of the SCG with identifier ℊ in the i-th session
$\Lambda_{u\left[S\left|R\right|G\right]}^{i_{k}}$	A one-time local authentication key (OLAK) for $M_{u}^{i_{k}}$	$\mathrm{RShift}\left(\cdot \right)$	The maximum number of iterations of a hash operation
⊕	Bitwise exclusive-OR operation		

**Table 2:** Probability of success of a cyber-attack as a function of the length of KMAC.

Length of KMAC (ϕ, bit)	Number of CAN Messages (n)	Collision Probability $\left(p\left(n;\phi \right),\;\%\right)$
**32**	**100**	**0.0001152511**
	**200**	**0.0004633319**
	1000	0.0116292153
	2000	0.0465320233
	**3000**	**0.1046840574**
**24**	**25**	**0.0017881242**
	**50**	**0.0073013096**
	100	0.0295000054
	200	0.1185433950
16	100	7.2785590519
	200	26.2108503279
8	100	99.999999928
	200	100

**Table 3:** Definition of a gateway’s message in the message set used.

Message ID (Hex)	Message	Size (bit)	Period (ms)	Deadline (ms)	From	To	Description
0	Session	8	1000	0	Gateway	All	Session Change

**Table 4:** Specification of hardware and software used in our experiment.

Tool	Model	Note
Microcontroller	Freescale S12FX	40 MHz and 512 KB
Emulator	USB S08/HCS12 BDM Multilink	-
Compiler	Code Warrior	For Freescale MCU
Software	CANoe v8.5	CAN Network simulator, CAPL, run on a system with an i7-7700 (4.20 GHz) Intel CPU and 16 G RAM
Connector	VN1630A	Interface device

**Table 5:** Mean time taken to perform hash functions one time in Freescale S12XF.

**Function**	**Processing Time (ms)**
SHA-256	5.3410
SHA-1	3.3328
mCRC32	0.0177

**Table 6:** Detection performance against brute-force attacks as a function of the bus load rate.

Bus load rate	58.7%	61%	63.3%	65.7%
Total number of CAN messages including brute-force attacks	221,193	230,426	238,686	247,682
Attack rate ^1^	3.9%	7.5%	10.8%	13.9%
Detection rate in TTI-AD ^1^	38.8%	45.0%	60.0%	70.3%
Detection rate in SHC-AD ^1^	61.2%	55.0%	40.0%	29.7%
False positive rate	0%	0%	0%	0%

^1^ Rounded to the first decimal place.

**Table 7:** Detection performance against delayed-replay attacks as a function of the bus load rate.

Bus load rate	56.32%	61.1%	63.1%	65.56%
Total number of CAN messages including delayed-replay attacks	212,172	230,214	236,282	245,240
Attack rate ^1^	0.1%	7.9%	10.4%	13.6%
Detection rate in TTI-AD ^1^	100%	1.0%	8.4%	30.8%
Detection rate in SHC-AD ^1^	0%	99.0%	91.6%	69.2%
False positive rate	0%	0%	0%	0%

^1^ Rounded to the first decimal place.

**Table 8:** Detection performance against cyber-attacks as a function of the bus load rate.

Bus load rate	66.93%	
Total number of CAN messages including cyber-attacks	252,974	
Attack type	Brute-force attacks	Delayed-replay attacks
Attack rate ^1^	6.78%	8.97%
Detection rate in TTI-AD ^1^	68.70%	20.03%
Detection rate in SHC-AD ^1^	31.30%	79.97%
False positive rate	0%	0%

^1^ Rounded to the first decimal place.
